# Thermally Modulated Microfluidic Fabrication of Phase-Tunable Cs_4_PbBr_6_/CsPbBr_3_ Hybrid Perovskite Nanocrystals for White Light-Emitting Diodes

**DOI:** 10.3390/nano16150962

**Published:** 2026-08-05

**Authors:** Yunhao Ning, Chuantong Cheng, Shuo Guan, Bao Zhang, Tuanning Liu, Di Shi, Wenqiang Liu, Beiju Huang

**Affiliations:** 1 State Key Laboratory of Optoelectronic Materials and Devices, Institute of Semiconductors, Chinese Academy of Sciences, Beijing 100083, China; ningyunhao9@163.com; 2 College of Materials Science and Optoelectronic Technology, University of Chinese Academy of Sciences, Beijing 100049, China; 3 School of Chemical Engineering and Technology, Tianjin University, Tianjin 300350, China; guanshuogs@126.com; 4 School of Optoelectronic Engineering, Henan Normal University, Xinxiang 453007, China; 2020001@htu.edu.cn (T.L.); 2522283047@stu.htu.edu.cn (D.S.)

**Keywords:** perovskite nanocrystals, Cs_4_PbBr_6_, CsPbBr_3_, microfluidic system, white light-emitting diodes

## Abstract

All inorganic CsPbBr_3_ perovskite nanocrystals (NCs) exhibit outstanding luminescence for optoelectronics, yet poor environmental stability severely restricts their practical deployment. As a stable derivative phase, Cs_4_PbBr_6_ can effectively improve structural stability. Nevertheless, the rational fabrication of high-quality Cs_4_PbBr_6_/CsPbBr_3_ hybrid NCs remains challenging owing to the lack of straightforward and scalable synthetic strategies. To overcome these hurdles, we synthesize well-defined Cs_4_PbBr_6_/CsPbBr_3_ hybrid NCs via a temperature-controllable continuous-flow microfluidic route. This platform precisely modulates phase composition via systematic temperature tuning across a range of 110–170 °C, producing distinct compositions from Cs_4_PbBr_6_-dominant to high-purity CsPbBr_3_. A direct correlation was elucidated between temperature-induced phase transformation and optical properties. The NCs synthesized at 130 °C exhibited a high photoluminescence quantum yield of 96.24% and bright 521 nm green emission. These NCs were successfully integrated into white light-emitting diodes incorporating a 478 nm blue excitation chip and K_2_SiF_6_:Mn^4+^ red phosphor, which demonstrated excellent color performance with a luminous efficiency of 86.3 lm W^−1^ and Commission Internationale de l’Éclairage coordinates of (0.2991, 0.3784). This work highlights the potential of continuous-flow microfluidics for precise phase modulation and scalable production of high-quality perovskite NCs, offering a viable route for advanced optoelectronic applications.

## 1. Introduction

Lead halide perovskite nanocrystals (NCs), exemplified by CsPbBr_3_, have garnered extensive research interest due to their exceptional optical and electronic properties, positioning them as ideal candidates for diverse optoelectronic applications [1,2,3,4,5]. Beyond the well-established CsPbBr_3_ phase, stoichiometric derivatives like Cs_4_PbBr_6_ have also emerged as noteworthy materials, owing to their distinct crystal structures and unique physicochemical characteristics [6,7,8,9,10]. Recent investigations have revealed that composite CsPbBr_3_/Cs_4_PbBr_6_ hybrid NCs exhibit enhanced luminescence and stability compared to their single-phase counterparts [11]. However, the controllable synthesis of these sophisticated hybrid structures presents a significant challenge with conventional batch methods, thus highlighting a critical need for novel synthetic strategies.

Despite the growing interest in Cs_4_PbBr_6_ and CsPbBr_3_ hybrid perovskite systems [12,13,14], the controlled modulation of phase transformation remains a significant obstacle for their scalable production and widespread application [15]. Theoretical studies indicate that the evolution of Cs-Pb-Br phases is highly sensitive to reaction parameters [16,17]. However, conventional batch synthesis methods struggle to provide the fine, continuous control required to tune this process effectively, frequently leading to mixed phases, poor reproducibility, and low yields. Recently, various post-synthetic temperature-controlled annealing strategies [18,19,20,21,22] have been developed to regulate the phase composition and crystalline structure of Cs_4_PbBr_6_/CsPbBr_3_ mixed-phase perovskites. These studies have provided valuable insights into temperature-dependent phase evolution and demonstrated the feasibility of thermal modulation for phase engineering. Nevertheless, achieving rapid and spatially uniform phase control remains challenging in conventional batch-based systems due to limited heat transfer and diffusion-controlled processes. Microfluidic reactors, with their inherent ability to maintain uniform thermal environments and precise parameter control [23,24,25,26,27], offer a compelling solution to these challenges. Nevertheless, existing microfluidic studies on Cs-Pb-Br derivatives have predominantly focused on static reaction conditions [28,29], lacking systematic investigations into how dynamic temperature gradients influence phase evolution. Critically, the ability to precisely tailor the phase composition of Cs_4_PbBr_6_/CsPbBr_3_ NCs via microfluidic platforms remains insufficiently explored, and the intricate link between phase structure and optical performance in these hybrid systems is still not fully elucidated.

Herein, we report a continuous-flow microfluidic approach for the controlled synthesis of Cs_4_PbBr_6_/CsPbBr_3_ hybrid perovskite NCs. By systematically modulating the reaction temperature within our microchannel reactor, we achieved unprecedented precise control over the Cs_4_PbBr_6_-to-CsPbBr_3_ phase transformation. This enabled the synthesis of a series of materials, ranging from mixed-phase composites to high-purity single-phase CsPbBr_3_. Specifically, we demonstrate that the phase evolution process is tunable solely through temperature control, highlighting the potential of the microfluidic platform for precise phase modulation. The optimized high-quality hybrid NCs were further integrated into white light-emitting diodes to verify their promising application potential in solid-state lighting and wide-color-gamut display devices.

## 2. Experimental Section

### 2.1. Material Preparation and Synthesis

Cesium carbonate (Cs_2_CO_3_, 99.99%), lead bromide (PbBr_2_, 99.99%), oleic acid (OA, 90%), oleylamine (OAm, 80–90%), 1-octadecene (ODE, ≥90%), n-hexane (≥98.0%), and methyl acetate (MeOAc, ≥99.0%) were purchased from Shanghai Aladdin Biochemical Technology Co. (Shanghai, China) and used as received without further purification.

The continuous synthesis of Cs_4_PbBr_6_/CsPbBr_3_ nanocrystals was performed on a custom-built three-stream microfluidic platform [30]. A polydimethylsiloxane microfluidic chip with a 200 μm channel diameter was employed, facilitating efficient molecular-level mixing of three precursor streams. Precursor solutions were prepared by separately loading oleate-capped cesium, lead bromide, and 1-octadecene into three syringes. These solutions were precisely injected into the microfluidic chip at constant flow rates using high-precision syringe pumps, enabling accurate control over the reaction residence time. The microfluidic chip was integrated with a temperature-controlled hot plate, maintaining reaction temperatures systematically from 110 to 170 °C to tune the phase evolution. Following reaction and mixing within the microchannel, the product stream was continuously collected from the chip outlet. The crude colloidal suspension was then subjected to purification: mixing with an equal volume of methyl acetate, followed by centrifugation at 5000 rpm for 20 min. The supernatant was discarded and the precipitate was redispersed in n-hexane. A second centrifugation under identical conditions yielded the precipitate, which was again redispersed in n-hexane. A final brief centrifugation (5000 rpm, 5 min) was performed to remove any residual colloidal impurities, resulting in monodisperse Cs_4_PbBr_6_/CsPbBr_3_ nanocrystal colloids.

### 2.2. Characterizations

Transmission electron microscopy (TEM) images were acquired using a TECNAI G2-20 microscope (Thermo Fisher Scientific, Hillsboro, OR, USA). Crystalline phases were identified by X-ray powder diffraction (XRD) using a Bruker D8 diffractometer (Bruker, Bremen, Germany). UV–visible (UV–vis) absorption spectra were recorded on a Shimadzu UV-2600 spectrometer (Shimadzu Corporation, Kyoto, Japan). Photoluminescence (PL), photoluminescence excitation (PLE), and time-resolved PL decay measurements were conducted using an Edinburgh FLS980 spectrophotometer (Edinburgh Instrument, Edinburgh, UK).

## 3. Results and Discussion

**Figure 1:**
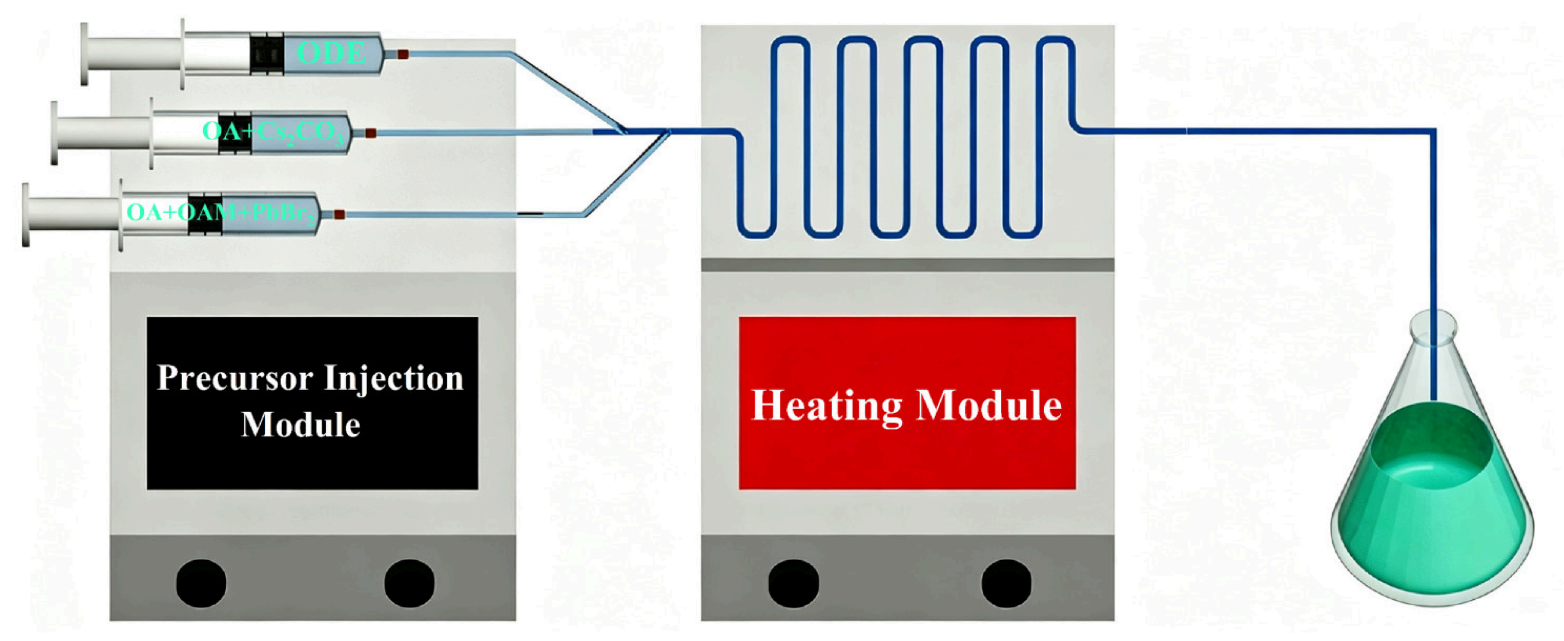
Schematic illustration of the temperature-regulated three-stream microfluidic platform for continuous synthesis of Cs_4_PbBr_6_/CsPbBr_3_ perovskite NCs.

CsPbBr_3_/Cs_4_PbBr_6_ NCs were continuously synthesized on a custom-built microfluidic platform (Figure 1). A split-stream feeding configuration facilitated precise stoichiometric control by adjusting individual syringe pumping rates, ensuring a fixed Cs-to-Pb and OA-to-OAM molar ratio of 2:1 for controlled nucleation. The precursor mixture was then subjected to thermal control within a serpentine microchannel, with four reaction temperatures (110, 130, 150, and 170 °C) evaluated for optimizing nanocrystal formation. The crude colloidal suspension was continuously harvested from the reactor outlet post-reaction.

**Figure 2:**
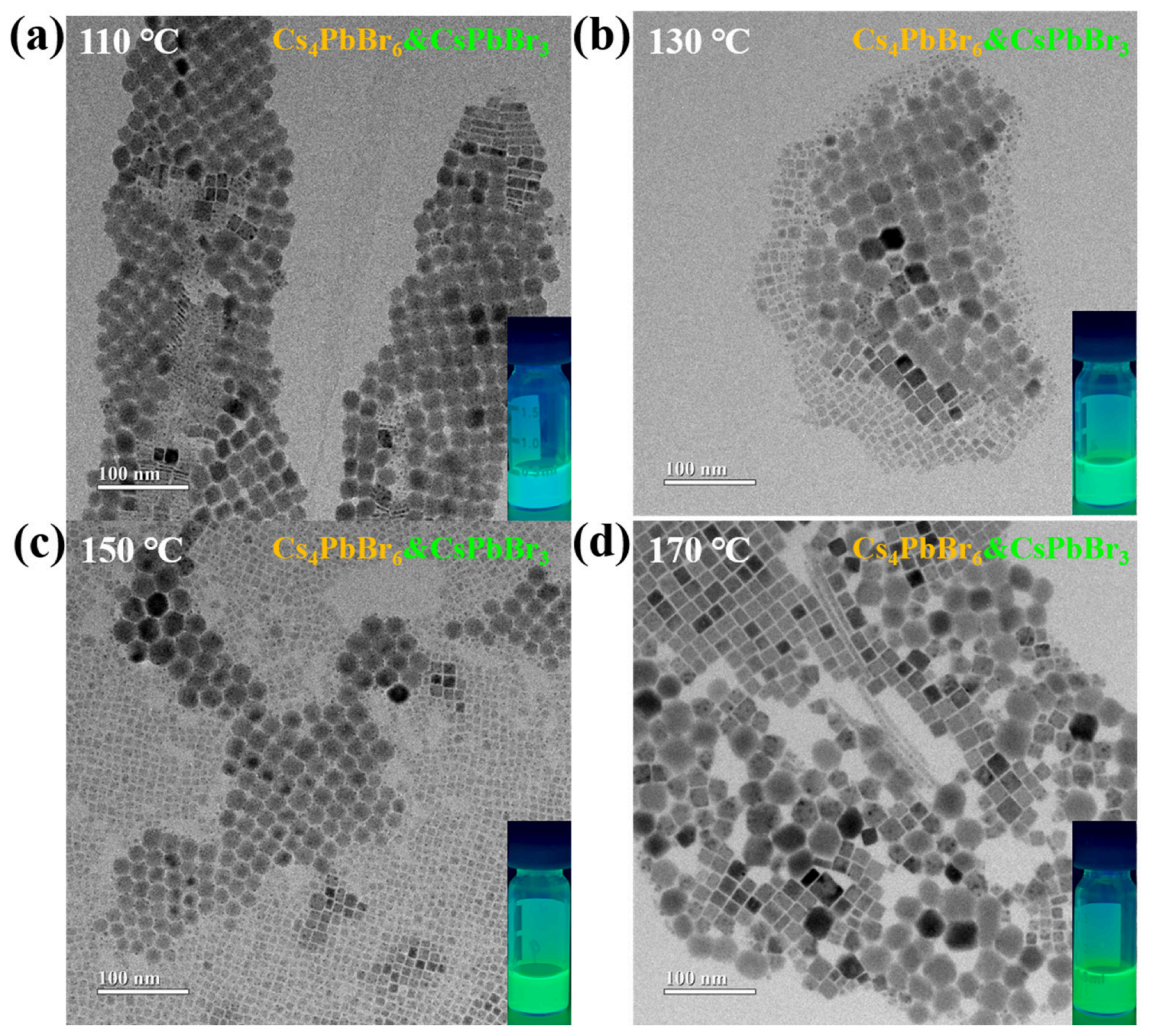
TEM images of Cs_4_PbBr_6_/CsPbBr_3_ hybrid NCs synthesized at distinct reaction temperatures. Panels (**a**–**d**) correspond to synthesis temperatures of 110 °C, 130 °C, 150 °C and 170 °C, respectively. Insets in each panel display the corresponding colloidal solutions under UV excitation, showing tunable green luminescence.

To observe the thermal-triggered crystal phase evolution, TEM characterizations were carried out on products obtained under four gradient temperatures. Figure 2 systematically revealed temperature-dependent phase transformation from Cs_4_PbBr_6_ to CsPbBr_3_. Synthesized samples exhibited distinct morphologies including spherical nanoparticles of hexagonal Cs_4_PbBr_6_ [31] and cubic CsPbBr_3_ [32], and such morphological differences show a direct correlation with reaction temperature. At 110 °C, insufficient thermal energy hindered Cs_4_PbBr_6_ lattice rearrangement [33] and suppressed the evolution toward CsPbBr_3_ formation, yielding primarily spherical Cs_4_PbBr_6_. Elevated temperatures within the microchannel accelerated precursor decomposition and atomic diffusion, facilitating in situ transformation. Notably, at 150 °C, precisely controlled thermal energy dramatically enhanced phase evolution toward CsPbBr_3_ formation, significantly boosting CsPbBr_3_ conversion efficiency and volume fraction. The inset images show bright green photoluminescence from all colloids in n-hexane under UV irradiation, confirming the CsPbBr_3_ luminescent properties. This controlled phase evolution demonstrates the effective thermal regulation capability of the microfluidic reactor for controlling phase transformation. To clarify the microstructure and phase distribution of the hybrid NCs, high-resolution transmission electron microscopy (HRTEM) analyses was performed (Figure S1). The NCs mainly consist of separately distributed CsPbBr_3_ and Cs_4_PbBr_6_ phases rather than a typical raisin-bread-like composite structure, in which one phase is randomly embedded within another. However, localized contact regions between CsPbBr_3_ and Cs_4_PbBr_6_ can be observed. In these contacted regions, distinct lattice fringes with d-spacings of 3.01 Å and 4.43 Å are identified, corresponding to the (200) plane of CsPbBr_3_ and the (113) plane of Cs_4_PbBr_6_, respectively. These lattice-resolved interfacial contacts provide direct evidence for the formation of nanoscale Cs_4_PbBr_6_/CsPbBr_3_ heterointerfaces, confirming the hybrid nature of the synthesized NCs.

**Figure 3:**
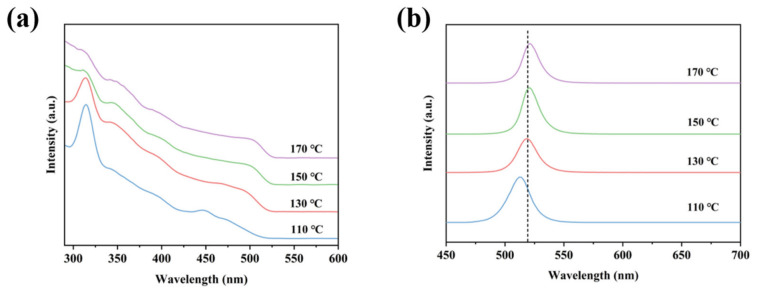
(**a**) UV–vis absorption spectra of samples obtained at 110 °C, 130 °C, 150 °C and 170 °C. (**b**) Corresponding steady-state photoluminescence spectra with a vertical dashed line marking the central emission peak position.

To further confirm the temperature-induced phase transition from optical absorption signatures, UV–vis absorption measurements were implemented for all hybrid samples. Figure 3a presents the UV–vis absorption spectra of Cs_4_PbBr_6_/CsPbBr_3_ hybrid NCs synthesized via microfluidic continuous flow. These spectra validate the Cs_4_PbBr_6_-to-CsPbBr_3_ solid-state phase transformation previously concluded from TEM characterization. At 110 °C, a dominant absorption peak at 315 nm confirms the prevalence of the Cs_4_PbBr_6_ crystalline phase [34], with negligible evidence of CsPbBr_3_ generation. As the reaction temperature is gradually elevated, the increased thermal energy accelerates lattice rearrangement [35,36] and atomic diffusion within the microchannel, thereby facilitating the in situ conversion of Cs_4_PbBr_6_ into CsPbBr_3_. This process is evidenced by a continuous growth in absorbance near 510 nm. The systematic evolution of these dual characteristic absorption bands [10] quantitatively demonstrates that elevated temperatures effectively boost the phase-transition kinetics from the intermediate Cs_4_PbBr_6_ to the target CsPbBr_3_ perovskite, further reinforcing that this microfluidic platform enables precise control over hybrid crystalline composition.

Figure 3b presents PL emission spectra of hybrid NCs. All four tested samples exhibit sharp and symmetric PL emission peaks centered around 520 nm, indicated by the vertical dotted line. This emission wavelength is highly consistent with the band-edge absorption feature [37,38] of CsPbBr_3_ observed in the preceding UV–vis spectra. As the synthetic temperature was stepwise-elevated from 110 to 170 °C, a gradual red-shift in the PL peak was observed. This slight red-shift may be attributed to the increasing content of CsPbBr_3_ within the hybrid nanocrystal system.

**Figure 4:**
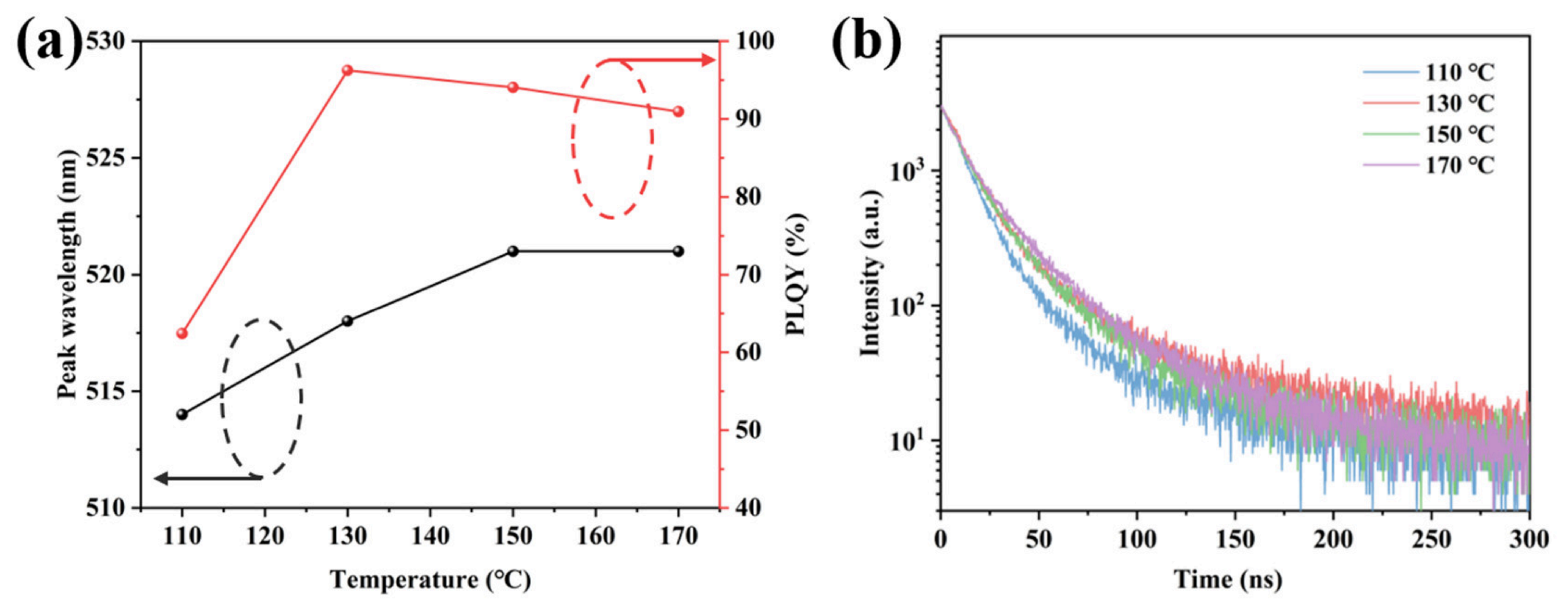
(**a**) Variation in PL emission peak wavelength and corresponding photoluminescence quantum yield (PLQY) for samples synthesized at temperatures ranging from 110 °C to 170 °C. (**b**) Time-resolved photoluminescence decay curves of Cs_4_PbBr_6_/CsPbBr_3_ hybrid NCs prepared at 110 °C, 130 °C, 150 °C and 170 °C.

To quantify the temperature modulation effect on luminescence performance, we tracked the variation in emission peak position and photoluminescence quantum yield across the full thermal gradient. Figure 4a plots the evolution of PL peak wavelength and PLQY with synthesis temperature. From 110 to 150 °C, PL emission wavelength gradually red-shifts from ~514 nm to ~521 nm, then remains nearly constant up to 170 °C. Conversely, PLQY sharply climbs from 62.41% at 110 °C to a peak of 96.24% at 130 °C. Beyond 130 °C, PLQY mildly decreases to 94.05% at 150 °C and 90.95% at 170 °C. This PLQY decline above 130 °C is likely due to excessive temperature causing mild nanoparticle agglomeration and new surface traps [39,40,41,42], increasing non-radiative losses. In addition to these structural effects, the superior PLQY at 130 °C can also be attributed to the optimized Cs_4_PbBr_6_/CsPbBr_3_ phase balance. The presence of the Cs_4_PbBr_6_ component provides effective interfacial passivation for CsPbBr_3_ domains, reducing surface-related defects and suppressing non-radiative recombination pathways. With further increasing temperature, the reduced Cs_4_PbBr_6_ contribution and enhanced crystal growth may weaken this passivation effect, resulting in increased defect-assisted recombination. TRPL measurements were employed to probe the recombination dynamics of the microfluidic-derived hybrid NCs (Figure 4b). Fitting the decay curves to a biexponential function [43,44] reveals that the fast component (*τ*_1_) represents non-radiative recombination, while the slow component (*τ*_2_) corresponds to radiative recombination. As detailed in the summarized Table 1, at 130 °C, the sample achieved the longest carrier lifetime *τ*_ave_ (25.21 ns), the highest radiative recombination rate constant k_r_ (0.038 ns^−1^), and the lowest non-radiative rate constant k_nr_ (0.0015 ns^−1^). These findings suggest that the precise thermal control enabled by our microfluidic platform fosters a favorable phase transformation, effectively mitigating surface trap states.

**Figure 5:**
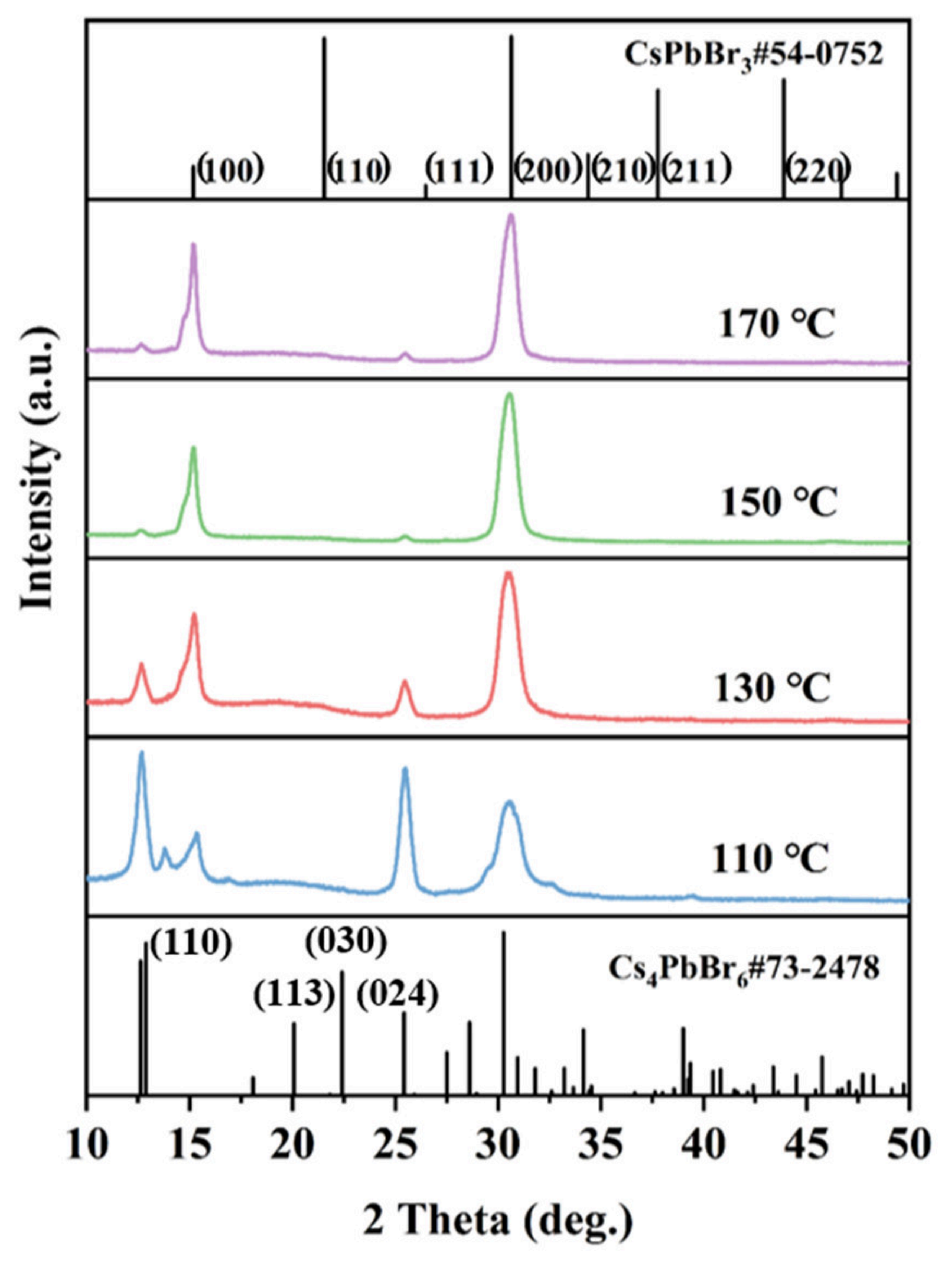
Stacked X-ray powder diffraction patterns of Cs_4_PbBr_6_/CsPbBr_3_ hybrid NCs synthesized at 110 °C, 130 °C, 150 °C and 170 °C. Standard reference diffraction peaks of pure Cs_4_PbBr_6_ (PDF #73-2478) and pure CsPbBr_3_ (PDF #54-0752) are displayed at the bottom and top of the plot, respectively, for phase identification.

To confirm the phase evolution and crystallinity of the microfluidically synthesized Cs_4_PbBr_6_/CsPbBr_3_ hybrid NCs, powder X-ray diffraction (XRD) patterns were recorded across the temperature range of 110–170 °C (Figure 5). These XRD results revealed a clear temperature-dependent phase transformation. At 110 °C, the pattern indicated a mixed phase comprising Cs_4_PbBr_6_ (with characteristic peaks at 12.6° and 25.4°) [45] and CsPbBr_3_ (displaying broadened peaks at 15.2° and 30.6°) [46], suggesting poor CsPbBr_3_ crystallinity at this temperature. Upon increasing temperature to 130 °C, Cs_4_PbBr_6_ signals diminished while CsPbBr_3_ peaks intensified and sharpened, signifying improved crystallinity. At 150–170 °C, Cs_4_PbBr_6_ signals nearly disappeared, and the patterns exclusively matched the standard CsPbBr_3_ reference, confirming high-purity perovskite formation. The consistent cubic CsPbBr_3_ (100)/(200) plane reflections across all temperatures, coupled with the temperature-dependent attenuation of Cs_4_PbBr_6_ peaks, demonstrate the controlled phase evolution driven by thermal energy. The temperature-dependent phase evolution is likely associated with a thermally regulated dissolution–recrystallization process during the microfluidic synthesis. In the solution-phase reaction environment, temperature influences precursor dissolution, Pb–Br intermediate conversion, and subsequent nucleation/growth kinetics, thereby regulating the phase equilibrium between Cs_4_PbBr_6_ and CsPbBr_3_. Previous studies have suggested that Cs-based lead halide perovskite phase evolution involves the formation and conversion of Pb–Br complexes, highlighting the critical role of solution-phase precursor chemistry in determining the final phase composition [47,48,49]. At lower temperatures, insufficient reaction kinetics may hinder precursor conversion and CsPbBr_3_ crystallization, resulting in relatively poor CsPbBr_3_ crystallinity. With optimized thermal energy at 130 °C, accelerated precursor conversion and controlled crystal growth facilitate the formation of highly crystalline CsPbBr_3_ domains. Further temperature increase promotes crystal growth and alters the Cs_4_PbBr_6_/CsPbBr_3_ phase equilibrium toward a CsPbBr_3_-dominant composition.

**Figure 6:**
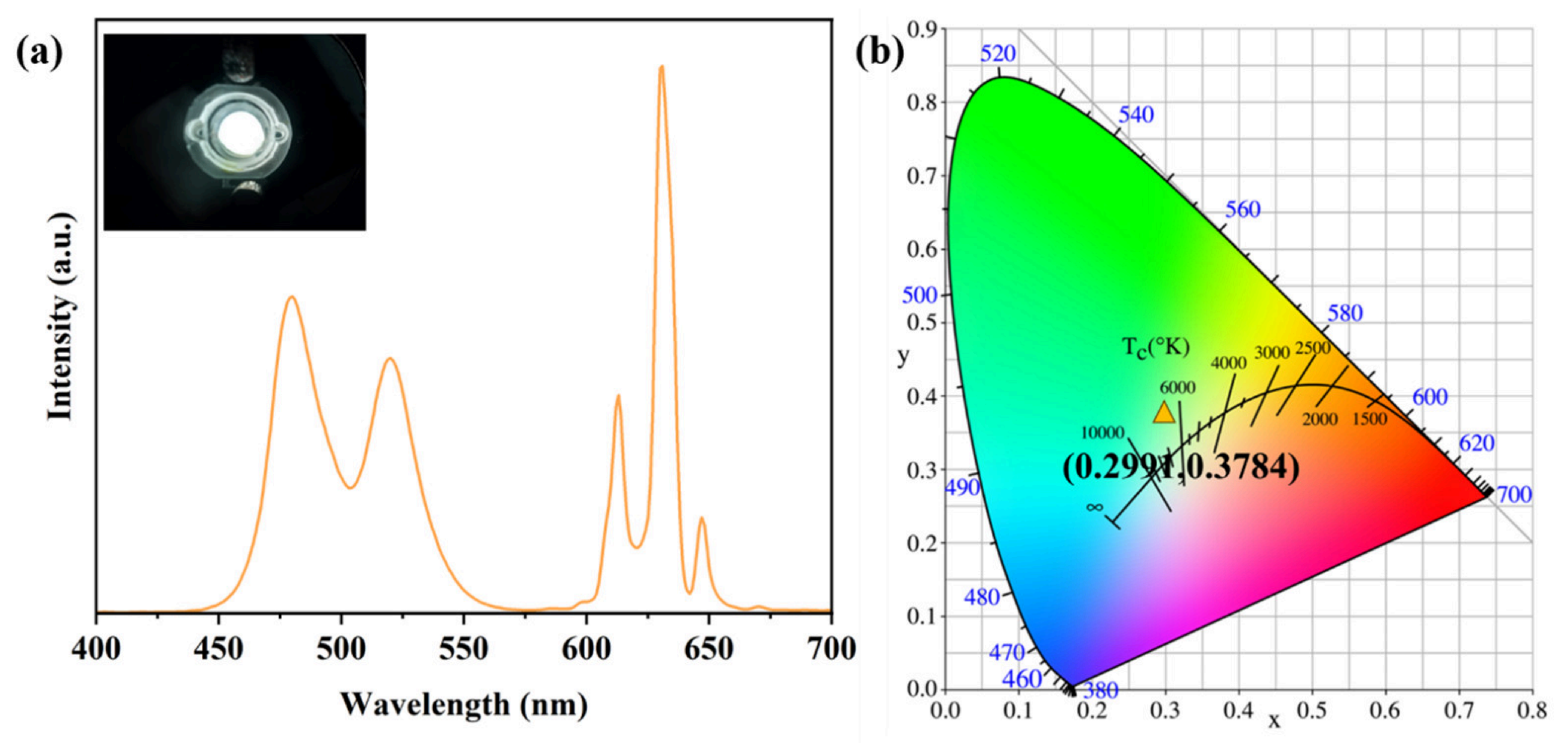
(**a**) Electroluminescence spectrum of the fabricated white light-emitting diode device, with an inset photograph showing the operating WLED chip. (**b**) Corresponding Commission Internationale de l’Éclairage chromaticity diagram.

Benefiting from the optimal luminescence performance of the 130 °C-synthesized composite NCs, we further explored their practical optoelectronic application as light-emitting phosphors. Recent studies have demonstrated that Cs_4_PbBr_6_/CsPbBr_3_ hybrid systems can achieve excellent LED performance through phase engineering and vacuum thermal evaporation strategies, highlighting the importance of precise phase control for optoelectronic applications [50,51,52]. In comparison, our continuous-flow microfluidic approach provides a solution-processable route for the synthesis of colloidal hybrid nanocrystals with tunable phase composition, offering an alternative strategy for phosphor-based LED applications. The synthesized CsPbBr_3_/Cs_4_PbBr_6_ hybrid NCs, exhibiting a high photoluminescence quantum yield of 96.24% and sharp green emission centered at ~520 nm, were employed as the green phosphor in a white light-emitting diode (WLED). This WLED device [53,54] was fabricated by integrating these NCs with a 478 nm blue excitation chip and a K_2_SiF_6_:Mn^4+^ red phosphor, resulting in a balanced spectrum for neutral white light emission (Figure 6a). The precise alignment of the green emission with the NCs’ PL peak ensured efficient conversion, contributing a strong green component. The resultant WLED demonstrated a luminous efficiency of 86.3 lm·W^−1^ and CIE coordinates of (0.2991, 0.3784) at 30 mA (Figure 6b). This high performance, enabled by continuous microfluidic synthesis, highlights the NCs’ potential for next-generation wide-color-gamut displays.

## 4. Conclusions

In conclusion, this work reports a temperature-regulated microfluidic strategy for the controlled synthesis of Cs_4_PbBr_6_/CsPbBr_3_ hybrid perovskite NCs with precisely tunable phase compositions. Our continuous-flow platform enables exceptional control over reaction conditions, allowing for systematic tuning of phase evolution from Cs_4_PbBr_6_ to high-quality CsPbBr_3_. Critically, we elucidated the direct correlation between temperature-induced phase transformation and the resulting optical properties of the composites. Our findings reveal that the enhanced luminescence performance is intrinsically linked to an optimized Cs_4_PbBr_6_ content. The optimized NCs were successfully fabricated into white light-emitting diodes. This work offers a viable route for controlled hybrid perovskite synthesis and highlights their potential for optoelectronic device applications.

## Figures and Tables

**Table 1:** Data summary of TRPL and PLQY spectra of samples synthesized at different temperature conditions.

Temperature (°C)	PLQY	*τ*_1_ (ns)	*τ*_2_ (ns)	*τ*_ave_ (ns)	*τ*_r_ (ns)	k_r_ (ns^−1^)	k_nr_ (ns^−1^)
110	62.41	13.02	95.33	20.66	33.11	0.030	0.018
130	96.24	14.61	78.68	25.21	26.20	0.038	0.0015
150	94.05	12.32	38.71	20.39	21.68	0.046	0.0029
170	90.95	10.98	32.96	21.71	23.87	0.042	0.0042

## Data Availability

The raw data supporting the conclusions of this article will be made available by the authors on request.

## Supplementary Materials

The following supporting information can be downloaded at https://www.mdpi.com/article/10.3390/nano16150962/s1, Figure S1: (a) Representative TEM image of the Cs_4_PbBr_6_/CsPbBr_3_ hybrid nanocrystals synthesized at 130 °C, showing a uniform morphological distribution. (b) HRTEM image of a single hybrid nanocrystal, revealing the intimately intergrown interface between the CsPbBr_3_ (200) plane (d = 3.01 Å) and the Cs_4_PbBr_6_ (113) plane (d = 4.43 Å). The clear lattice continuity indicates the formation of a nanoscale heterostructure.

## References

[1] Wang Y.K., Wan H., Teale S., Grater L., Zhao F., Zhang Z., Duan H.W., Imran M., Wang S.D., Hoogland S. (2024). Long-range order enabled stability in quantum dot light-emitting diodes. Nature.

[2] Kim S., Kim S.A., Park G.S., Kim E., Kim D.H., Lee S.C., Woo S.J., Jang Y., Kweon J.J., Kang S. (2026). Cold-injection synthesis of highly emissive perovskite nanocrystals. Nature.

[3] Zeng Q., Zhao Y., Park S., Zhou H., Shim H.J., Li T., Ryu J., Sung M.J., Chua X.W., Yoon E. (2026). A hierarchical shell locks and stabilizes perovskite nanocrystals with near-unity quantum yield. Science.

[4] Zheng S., Wang Z., Jiang N., Huang H., Wu X., Li D., Teng Q., Li J., Li C., Li J. (2024). Ultralow voltage-driven efficient and stable perovskite light-emitting diodes. Sci. Adv..

[5] Yoon S.S., Lee S.H., Lee M.J., Kim S.J., Ahn H., Park H.J., Shim J.W. (2026). Ionic Landscape Engineering via Perovskite Quantum Dots for Reliable and Energy-Efficient Perovskite Memristors. ACS Nano.

[6] Saidaminov M.I., Almutlaq J., Sarmah S., Dursun I., Zhumekenov A.A., Begum R., Pan J., Cho N., Mohammed O.F., Bakr O.M. (2016). Pure Cs_4_PbBr_6_: Highly Luminescent Zero-Dimensional Perovskite Solids. ACS Energy Lett..

[7] Liu Y., Wang X., Xie Q., Wang J., Yu W., Zhao L., Yang J., Wu D., Tang X., Tian S. (2025). Interface Engineering with Bifunctional Addictive for Efficient and Stable Blue Perovskite LED from In Situ Formed Cs_4_PbBr_6_ Heterostructured CsPbBr_3_ Quantum Dots. Laser Photonics Rev..

[8] Xia Z., Jiang J., Wang A., An D., Li Z., Zheng H., Yin Z., He G., You J., Zhang X. (2025). Overall Performance Improvement of Perovskite Green LEDs by CsPbBr_3_&Cs_4_PbBr_6_ Nanocrystals and Molecular Doping. Adv. Mater..

[9] Kang B., Biswas K. (2018). Exploring Polaronic, Excitonic Structures and Luminescence in Cs_4_PbBr_6_/CsPbBr_3_. J. Phys. Chem. Lett..

[10] Wu R., Gong S., Wang Q., Demontis V., Lai S., Matta S., Wu W., Marongiu D., Chen R., Saba M. (2025). Reversible Thermochromism in Cs_4_PbBr_6_ Microcrystals/CsPbBr_3_ Nanocrystals Based on the Synergistic Interaction between Cesium Ions and PbBr_6_ Octahedra. ACS Nano.

[11] Shi H., Huang J., Sun T., Yu H., Hu Y., Li X., Ma X., Yang W., Liu Z., Wang L. (2025). Modulating Crystallization of Perovskite Thin Films via Zero-Dimensional Cs_4_PbBr_6_ Nanocrystals. Adv. Funct. Mater..

[12] Kim H., Park J.H., Kim K., Lee D., Song M.H., Park J. (2022). Highly Emissive Blue Quantum Dots with Superior Thermal Stability via In Situ Surface Reconstruction of Mixed CsPbBr_3_-Cs_4_PbBr_6_ Nanocrystals. Adv. Sci..

[13] Lai W., Wu C., Han X. (2023). Continuous and controllable synthesis of Cs_x_Pb_y_Br_z_-based perovskite nanocrystals by a microfluidic system. Chem. Eng. J..

[14] Bazri B., Singh S., Dave K., Wei D.H., Liu R.S. (2025). Surface Trait-Dependent Photoreduction Products in CsPbBr_3_, Embedded Cs_4_PbBr_6_ Structure. Small.

[15] Chen X., Zhang F., Ge Y., Shi L., Huang S., Tang J., Lv Z., Zhang L., Zou B., Zhong H. (2018). Centimeter-Sized Cs_4_PbBr_6_ Crystals with Embedded CsPbBr_3_ Nanocrystals Showing Superior Photoluminescence: Nonstoichiometry Induced Transformation and Light-Emitting Applications. Adv. Funct. Mater..

[16] Yin J., Zhang Y., Bruno A., Soci C., Bakr O.M., Brédas J.-L., Mohammed O.F. (2017). Intrinsic Lead Ion Emissions in Zero-Dimensional Cs_4_PbBr_6_ Nanocrystals. ACS Energy Lett..

[17] Wang M., Zhang J., Su M., Zhang F., Yang B., Ji X., Zeng Q., Jia M., Ma Z., Chen X. (2025). Highly Bright and Stable Chiral CsPbBr_3_ Perovskite Nanocrystal Scintillators for High-Resolution X-ray Imaging. Nano Lett..

[18] Tan Y., Tian T., Chen H., Yang G., Yang M., Zhong J.X., Chen Z.R., He Y., Wu W.Q. (2026). Supramolecular Chloride Reservoirs Enable Homogeneous Halide Distribution and Near-Unity Blue Luminescence in Cs_4_PbBr_6_-CsPbBr_3_ Heterostructured Perovskites. Angew. Chem. Int. Ed. Engl..

[19] Pratama P.R., Phuaran V., Afif I.A., Pramata A.D., Agutaya J.K.C.N., Yoneda S., Inomata Y., Takafuji M., Akaishi Y., Kida T. (2026). Interfacial Ligand Density Regulates Solvent-Induced Phase Transformation in CsPbBr_3_/Cs_4_PbBr_6_ Nanocrystals. Chem. Mater..

[20] Saito H., Kihara K., Ikeuchi M., Yanagimoto S., Kubota T., Akiba K., Sannomiya T. (2025). Nano-light source generation by electron beam irradiation of CsPbBr_3_/Cs_4_PbBr_6_ composites. Appl. Phys. Lett..

[21] Wang X., Yin Q., Xu R., Gu Y., Huang X., Li M., Ma T., Chen Z., Chen J., Zeng H. (2024). Multiwavelength Temperature-Controlled Phase Modulator Based on Cs_4_PbBr_6_/CsPbBr_3_ Perovskite Crystals. ACS Photonics.

[22] Otero-Martinez C., Zaffalon M.L., Ivanov Y.P., Livakas N., Goldoni L., Divitini G., Bora S., Saleh G., Meinardi F., Fratelli A. (2024). Ultrasmall CsPbBr_3_ Blue Emissive Perovskite Quantum Dots Using K-Alloyed Cs_4_PbBr_6_ Nanocrystals as Precursors. ACS Energy Lett..

[23] Abdel-Latif K., Epps R.W., Kerr C.B., Papa C.M., Castellano F.N., Abolhasani M. (2019). Facile Room-Temperature Anion Exchange Reactions of Inorganic Perovskite Quantum Dots Enabled by a Modular Microfluidic Platform. Adv. Funct. Mater..

[24] Lignos I., Maceiczyk R.M., Kovalenko M.V., Stavrakis S. (2019). Tracking the Fluorescence Lifetimes of Cesium Lead Halide Perovskite Nanocrystals During Their Synthesis Using a Fully Automated Optofluidic Platform. Chem. Mater..

[25] Jha P., Mukhin N., Ghorai A., Morshedian H., Canty R.B., Delgado-Licona F., Brown E.E., Pyrch A.J., Castellano F.N., Abolhasani M. (2025). Photo-Induced Bandgap Engineering of Metal Halide Perovskite Quantum Dots in Flow. Adv. Mater..

[26] Yuan J., Diao J., Zheng Y., Hao J., Sun B., Zhao G., Li X., He G., Jiang X. (2025). Hierarchical Microfluidic Epitaxy of Sn^4+^-Stabilized Core–Shell Perovskites with Lead-Free Double Perovskite Shells for Highly Efficient and Ultra-Stable Emission. Adv. Funct. Mater..

[27] Chen G., Zhu X., Xing C., Wang Y., Xu X., Bao J., Huang J., Zhao Y., Wang X., Zhou X. (2022). Machine Learning-Assisted Microfluidic Synthesis of Perovskite Quantum Dots. Adv. Photonics Res..

[28] Bao Z., Tseng Y.J., You W., Zheng W., Chen X., Mahlik S., Lazarowska A., Lesniewski T., Grinberg M., Ma C. (2020). Efficient Luminescence from CsPbBr_3_ Nanoparticles Embedded in Cs_4_PbBr_6_. J. Phys. Chem. Lett..

[29] Xu Y., Huang B., Liu B., Li S., Zhang H., Yang H., Xu R. (2025). CsPbBr_3_ nanocrystals experience step temperature variation in flow: Morphology evolution and optical properties. J. Alloys Compd..

[30] Ning Y., Guan S., Cheng C., Zhang B., Qin B., Huang B. (2025). Microfluidic synthesis of monodispersed sharp emitting perovskite CsPbBr_3_ quantum dots via multidimensional parameterization. J. Mater. Chem. C.

[31] Chen Y., Zhang X., Jiang J., Chen G., Zhou K., Zhang X., Li F., Yuan C., Bao J., Xu X. (2024). Microfluidic synthesis of hollow CsPbBr_3_ perovskite nanocrystals through the nanoscale Kirkendall effect. Nano Res..

[32] Liu W., Qi Z., Liu T., Zhang Y. (2025). Fluoride Ion Passivation of CsPbBr_3_ Nanocrystals at Room Temperature for Highly Efficient and Stable White Light-Emitting Diodes. ACS Appl. Mater. Interfaces.

[33] Herrera Mondragon A., Gonzalez Rodriguez R., Hurley N., Varghese S., Jiang Y., Squires B., Cheng M., Davis B., Jiang Q., Mortazavi M. (2024). Forster Resonance Energy Transfer and Enhanced Emission in Cs_4_PbBr_6_ Nanocrystals Encapsulated in Silicon Nano-Sheets for Perovskite Light Emitting Diode Applications. Nanomaterials.

[34] Zhou Q., Zhao X., Jiang Y., Zhou J., Liu Z., Pan K. (2025). Aspect Ratio-Tunable CsPbBr_3_ Nanorods from Cs_4_PbBr_6_ at the Oil–Water Interface with Enhanced Stability via Ligand Treatment. ACS Appl. Nano Mater..

[35] Ye Q., Hu W., Zhu J., Cai Z., Zhang H., Dong T., Yu B., Chen F., Wei X., Yao B. (2024). TOPO-Suppressing charge recombination in a methylammonium-free wide-bandgap perovskite film for high-performance and stable perovskite solar cells. Energy Environ. Sci..

[36] Dong T., Shen C., Yu B., Zhao S., Wu H., Ding C., Shi B., Cai Z., Hu W., Shi B. (2025). Minimizing Solvent Residues in CsPbI_1.5_Br_1.5_ Perovskite Films for Efficient Ultra-Wide Bandgap Solar Cells. Carbon Neutraliz..

[37] Xu J., Huang W., Li P., Onken D.R., Dun C., Guo Y., Ucer K.B., Lu C., Wang H., Geyer S.M. (2017). Imbedded Nanocrystals of CsPbBr_3_ in Cs4 PbBr_6_: Kinetics, Enhanced Oscillator Strength, and Application in Light-Emitting Diodes. Adv. Mater..

[38] Hou L., Ringstrom R., Maurer A.B., Abrahamsson M., Andreasson J., Albinsson B. (2022). Optically Switchable NIR Photoluminescence of PbS Semiconducting Nanocrystals using Diarylethene Photoswitches. J. Am. Chem. Soc..

[39] Wang P., Yang K., Zhang L., Dai X., Ye Z., He H., Fan C. (2025). Suppressing Thermal- and Light-Induced Photoluminescence Quenching in Perovskite Quantum Dots Through Fluoride Post-Treatment. Small.

[40] Yang B., Zu Y., Wen X., Ji X., Wang M., Zeng Q., Liu Q., Lian L., Jia M., Chen X. (2025). Enhanced Stability of Cyan CsPb(Br/Cl)_3_ Perovskite Nanocrystals via Ligand Crosslinking for Underwater Wireless Optical Communication. Laser Photonics Rev..

[41] Jiang Q., Tong J., Xian Y., Kerner R.A., Dunfield S.P., Xiao C., Scheidt R.A., Kuciauskas D., Wang X., Hautzinger M.P. (2022). Surface reaction for efficient and stable inverted perovskite solar cells. Nature.

[42] Nughays R.O., Almasabi K., Nematulloev S., Wang L., Bian T., Nadinov I., Irziqat B., Harrison G.T., Fatayer S., Yin J. (2024). Mapping Surface-Defect and Ions Migration in Mixed-Cation Perovskite Crystals. Adv. Sci..

[43] Fang X., Huang Q., Lu R., Tang Y., Lu Y., Zheng B., Yan D., Yuan Z., Ahijado Guzmán R., Ye W. (2026). Chiral Perovskites: Controlled Synthesis and Photonic Device Applications. ACS Photonics.

[44] Jiang X., Huang H., Wang K., Dai X., Ye Z., He H., Fan C. (2025). Ultrastable Silica-Confined CsSnI_3_ Perovskite Nanocrystals for Noncontact Near-Infrared Light Communication and Sunlight-Thermal-Electric Conversion. ACS Nano.

[45] Li J., Liao K., Zhang J., Yuan F. (2025). High-Efficiency Ultrabroadband Warm White Light Emission from a Single-Component Lead-Free Perovskite with Record-Low Blue-Violet Content. Adv. Funct. Mater..

[46] Rao L., Sun B., Liu Y., Zhong G., Wen M., Zhang J., Fu T., Wang S., Wang F., Niu X. (2023). Highly Stable and Photoluminescent CsPbBr_3_/Cs_4_PbBr_6_ Composites for White-Light-Emitting Diodes and Visible Light Communication. Nanomaterials.

[47] Xie C., Zhang X., Chen H.S., Yang P. (2024). Highly Bright and Stable CsPbX_3_@Cs_4_PbX_6_ Hexagonal Nanoarchitectonics Created by Controlling Dissolution-Recrystallization of CsPbX_3_ Nanomaterials. Small.

[48] Hui J., Jiang Y., Gokcinar O.O., Tang J., Yu Q., Zhang M., Yu K. (2020). Unveiling the two-step formation pathway of Cs_4_PbBr_6_ nanocrystals. Chem. Mater..

[49] Liu Z., Bekenstein Y., Ye X., Nguyen S.C., Swabeck J., Zhang D., Lee S.-T., Yang P., Ma W., Alivisatos A.P. (2017). Ligand mediated transformation of cesium lead bromide perovskite nanocrystals to lead depleted Cs_4_PbBr_6_ nanocrystals. J. Am. Chem. Soc..

[50] Du P., Li J., Wang L., Sun L., Wang X., Xu X., Yang L., Pang J., Liang W., Luo J. (2021). Efficient and large-area all vacuum-deposited perovskite light-emitting diodes via spatial confinement. Nat. Commun..

[51] Hsieh C.-A., Tan G.-H., Chuang Y.-T., Lin H.-C., Lai P.-T., Jan P.-E., Chen B.-H., Lu C.-H., Yang S.-D., Hsiao K.-Y. (2023). Vacuum-Deposited Inorganic Perovskite Light-Emitting Diodes with External Quantum Efficiency Exceeding 10% via Composition and Crystallinity Manipulation of Emission Layer under High Vacuum. Adv. Sci..

[52] Jung N.E., Kang D., Park J., Kim K., Kim J., Kim J., Lee C.-H., Lee G.-D., Blumstengel S., Morales N.Z. (2026). Precisely controlled organic halide evaporation for efficient vacuum-deposited perovskite light-emitting diodes. Appl. Phys. Rev..

[53] Xu Y., Hu X., Chen H., Tang H., Hu Q., Chen T., Jiang W., Wang L., Jiang W. (2022). In situ passivation of Pb^0^ traps by fluoride acid-based ionic liquids enables enhanced emission and stability of CsPbBr_3_ nanocrystals for efficient white light-emitting diodes. Nanoscale.

[54] Padhiar M.A., Ji Y., Wang J., Khan N.Z., Xiong M., Wang S. (2025). ZrBr_4_-Mediated Phase Engineering in CsPbBr_3_ for Enhanced Operational Stability of White-Light-Emitting Diodes. Nanomaterials.

