# Supplementary material for: Thermally Modulated Microfluidic Fabrication of Phase-Tunable Cs4PbBr6/CsPbBr3 Hybrid Perovskite Nanocrystals for White Light-Emitting Diodes
Source: Nanomaterials (Basel). 2026 Aug 5;16(15):962. doi: 10.3390/nano16150962 (PMC13468539; doi:10.3390/nano16150962)
Supplement: Supplementary file 1 [file nanomaterials-16-00962-s001.zip › nanomaterials-4462098-supplementary.pdf]

Thermally Modulated Microfluidic Fabrication of Phase-Tunable $Cs_4PbBr_6/CsPbBr_3$ Hybrid Perovskite Nanocrystals for White Light-Emitting Diodes

Yunhao Ning [1], Chuantong Cheng [1,2,*], Shuo Guan [3], Bao Zhang [3,*], Tuanning Liu [4], Di Shi [4], Wenqiang Liu [4,*] and Beiju Huang [1,2,*]

[1] State Key Laboratory of Optoelectronic Materials and Devices, Institute of Semiconductors, Chinese Academy of Sciences, Beijing 100083, China; ningyunhao9@163.com

[2] College of Materials Science and Optoelectronic Technology, University of Chinese Academy of Sciences, Beijing 100049, China

[3] School of Chemical Engineering and Technology, Tianjin University, Tianjin 300350, China; guanshuogs@126.com

[4] School of Optoelectronic Engineering, Henan Normal University, Xinxiang, Henan 453007, China; 2522283047@stu.htu.edu.cn (D.S.); 2020001@htu.edu.cn (T.L.)

[*] Correspondence: chengchuantong@semi.ac.cn (C.C.); baozhang@tju.edu.cn (B.Z.); 2020131@htu.edu.cn (W.L); bjhuang@semi.ac.cn(B.H.);

Tel.: +010-82304531 (C.C.)

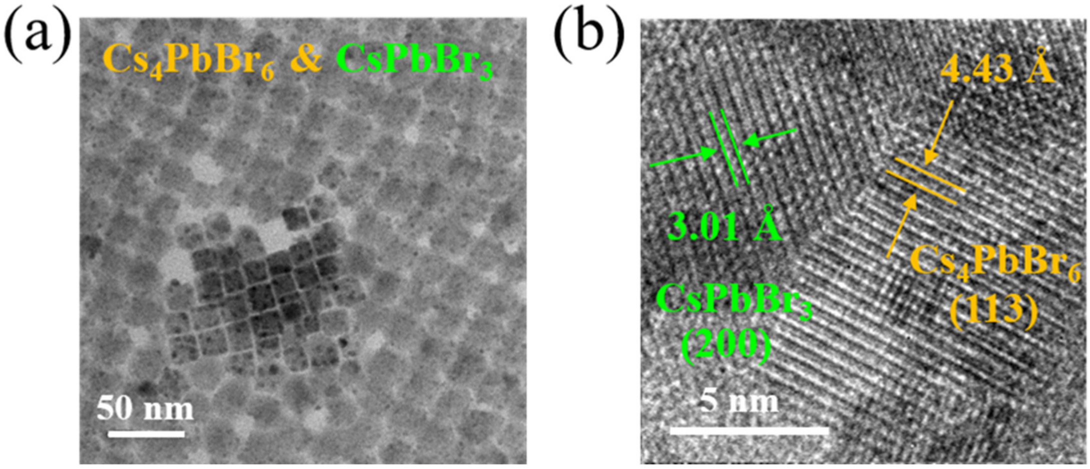

Figure S1. (a) Representative TEM image of the Cs_4PbBr_6/$CsPbBr_3$ hybrid nanocrystals synthesized at 130 °C, showing a uniform morphological distribution. (b) HRTEM image of a single hybrid nanocrystal, revealing the intimately intergrown interface between the $CsPbBr_3$ (200) plane (d = 3.01 Å) and the Cs_4PbBr_6 (113) plane (d = 4.43 Å). The clear lattice continuity indicates the formation of a nanoscale heterostructure.
